# Voluntary adoption of social welfare-enhancing behavior: Mask-wearing in Spain during the COVID-19 outbreak

**DOI:** 10.1371/journal.pone.0242764

**Published:** 2020-12-01

**Authors:** Joan Barceló, Greg Chih-Hsin Sheen

**Affiliations:** Division of Social Science, New York University Abu Dhabi, Abu Dhabi, United Arab Emirates; Columbia University, UNITED STATES

## Abstract

With the spread of COVID-19, more countries now recommend their citizens to wear facemasks in public. The uptake of facemasks, however, remains far from universal in countries where this practice lacks cultural roots. In this paper, we aim to identify the barriers to mask-wearing in Spain, a country with no mask-wearing culture. We conduct one of the first nationally representative surveys (n = 4,000) about this unprecedented public health emergency and identify the profile of citizens who are more resistant to face-masking: young, educated, unconcerned with being infected, and with an introverted personality. Our results further indicate a positive correlation between a social norm of mask-wearing and mask uptake and demonstrate that uptake of facemasks is especially high among the elderly living in localities where mask-wearing behavior is popular. These results are robust when controlling for respondents’ demographics, time spent at home, and occupation fixed effects. Our findings can be useful for policymakers to devise effective programs for improving public compliance.

## Introduction

**Fig 1:**
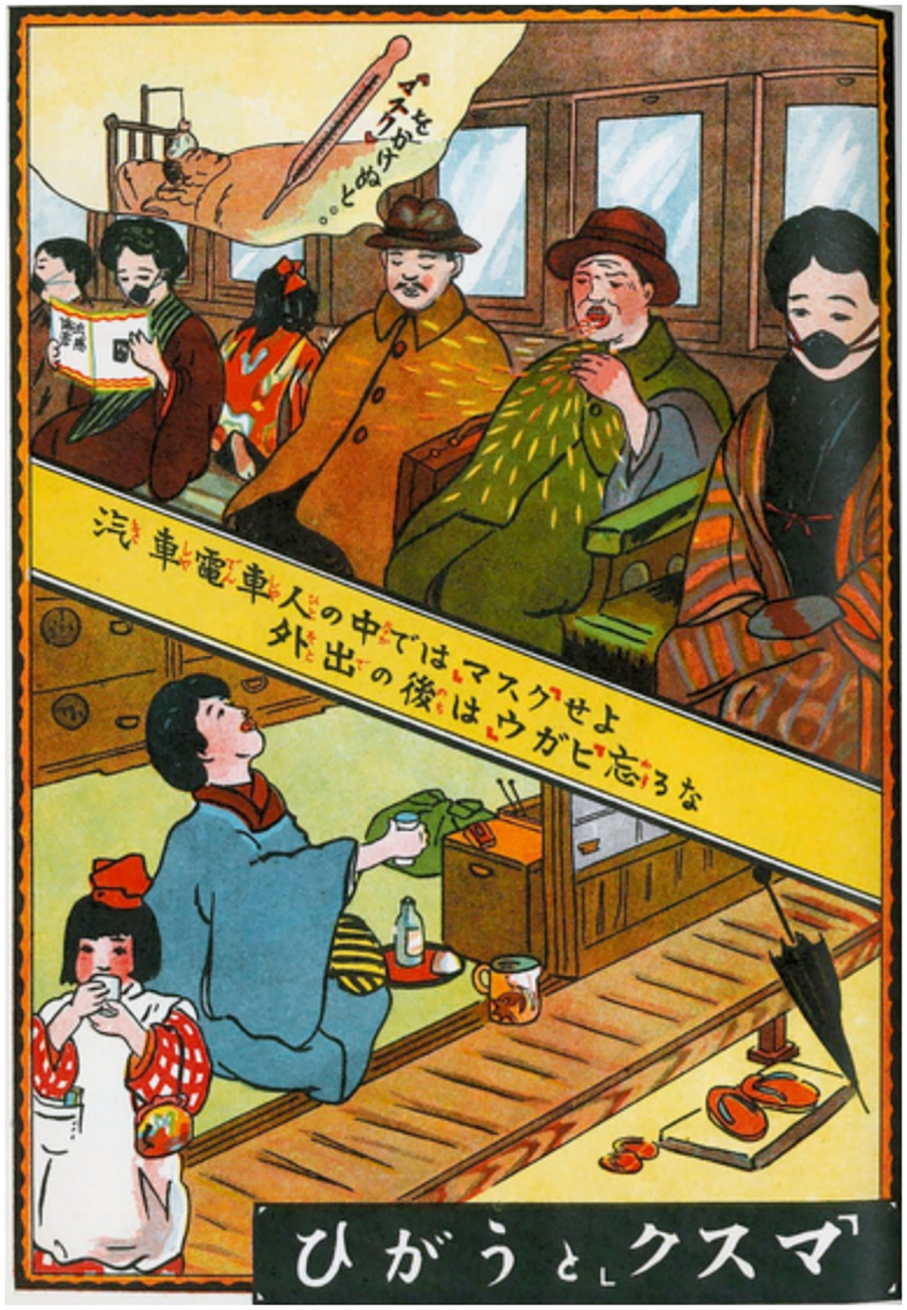
A poster promoting mask-wearing in Japan during the Spanish flu outbreak. Source: Asahi Shimbun https://digital.asahi.com/articles/ASN4S4CYPN4FUTIL01M.html.

For preventing the spread of infectious diseases, probably since the 1918 outbreak of the Spanish flu, the century-old debate about whether the public should wear a facial mask has begun. During that outbreak, in some places around the world, such as Japan, the wearing of a layered gauze mask over the mouth in public were recommended (Fig 1), and the practice of mask-wearing has since become a custom [1]. After the 2003 outbreak of Severe Acute Respiratory Syndrome (SARS), mask-wearing is widely adopted in East Asia as a form of non-pharmaceutical intervention for reducing transmission of respiratory infection. However, unlike hand-washing, which is universally considered to be the most important measure for preventing infectious disease, mask-wearing enjoys a mixed reception across countries until today.

Despite the growing evidence of the effectiveness of face mask use against the transmission of respiratory viruses [2–5], there have been dramatic differences in policy recommendations and public acceptability of mask-wearing across countries [6, 7]. Early in the COVID-19 outbreak, East Asian governments recommended, or even mandated, their citizens to wear a protective mask in public, for example, on January 22, Wuhan residents were ordered to wear facemasks in public spaces (see [8]), and citizens ubiquitously adopted this practice. In contrast, many other health authorities around the world, including the Centers for Disease Control and Prevention of the United States, suggested only healthcare workers and sick people should wear masks. For a lively debate about whether the public should wear face masks for preventing COVID-19, see [9]. In early April, many of these public health authorities have shifted their early advice and are now increasingly encouraging citizens to wear masks in the community [10]. Nevertheless, the uptake of facemasks remains far from universal in countries where this practice lacks cultural roots.

As the COVID-19 continues to spread, governments around the world, including the US and the UK, debate whether to recommend or mandate the use of face mask in public. Some other countries, such as Canada and UAE, have already made mask-wearing in public mandatory. While direct policy action is important, Condon and Sinha [11]’s Mexico City-based study during an H1N1 influenza outbreak shows that making mask-wearing mandatory has little impact on compliance in the absence of strong enforcement mechanisms. Hence, in most countries and geographic areas, mask-wearing behavior will ultimately depend on citizens’ voluntary uptake.

In this paper, we aim to identify the barriers to mask-wearing behavior in a country where wearing a face mask has been an outlier behavior until the COVID-19 outbreak. Answers to this issue are far from comprehensive in the literature. Note that the effectiveness of facemasks, on the other hand, differs greatly with factors, such as types of mask, mask materials, geographical and social-economic characteristics of a society, see [12] for a detailed review.

In a review of 25 studies on uptake of masks against respiratory infections at mass gatherings, Barasheed et al. [2] found that only three studies evaluated the reasons of using facemask and “discomfort and difficulty in breathing were the most reported reasons for not wearing facemask” (108). Even extending our focus beyond those studies related to “mass gatherings”, there are only few more relevant pieces investigating the uptake of facial masks. Generally speaking, earlier work has investigated three sets of predictors of mask-wearing behavior: demographics, threat perceptions, and negative emotions. It is worth noting that, recently, a newly designed and easier to use type of masks is invented, so we should worry less about comfort and mask-usage as hurdles to mask-wearing. See Alenezi et al. [13] for a detailed discussion.

We contribute to this literature by investigating the key predictors of mask-wearing behavior using evidence from a nationally representative survey conducted in Spain in the early stages of the COVID-19 pandemic at a time when wearing a facemask was not yet mandatory. We empirically investigate four sets of predictors: demographic characteristics, risk perceptions of contracting the virus, personality traits, and social acceptability of mask-wearing. As such, some of our investigations and results are comparable to the findings in the literature; many have never been documented before.

First, Jordan et al. [14], Capraro and Barceló [15] and Haischer et al. [16] report significant gender and age differences in mask-wearing behavior with older people and women being more likely to wear a mask than younger people and men in three different convenient samples from the United States. While we do not find a significant effect of gender, we replicate earlier findings and show that older cohorts are particularly more likely to wear a protective facemask. Beyond analyzing the effects of gender and age in our Spanish sample, we contribute to earlier work by studying the impact of educational attainment. In a Hong Kong study during an H1N1 influenza epidemic, Lau et al. [17] found that highly educated citizens were more likely to wear a mask. At the time of the survey in Spain, however, health experts and authorities had provided mixed advice on the role of facemasks. Hence, our results depart from Lau et al.’s [17] findings and reveal that respondents who are better educated were consistently *less* likely to wear a protective mask in the early stages of the pandemics in Spain.

Second, risk perceptions to a disease-related threat has been found a key predictor of mask-wearing behavior in previous studies from Norway [18] during an influenza, United Kingdom [19] on the swine flu outbreak, Hong Kong [17], and the United States [14] during the COVID-19. We replicate these earlier works and find that the relationship between risk perceptions and mask-wearing behavior generalizes to a representative survey of Spanish people in the context of the COVID-19 pandemics.

Third, prior work had suggested that negative emotions of wearing facemasks are associated with how other perceive the mask-wearer. For instance, Capraro and Barceló [15] show that people are less likely to wear a facemask if they agree with the statements: “wearing a face covering is shameful”, “wearing a face covering is a sign of weakness”, and “the stigma attached to wearing a face covering is preventing me from wearing one as often as I should.” A major contribution of our study is the establishment of an association between the Big-Five personality traits and the voluntary adoption of a social welfare-enhancing behavior. While the Big Five personality traits have been associated with numerous social [20], political [21, 22], and health behavior [23, 24], this is the first paper to empirically investigate the role of the Big Five in wearing facial masks. Given the prior correlations established by Capraro and Barceló [15], we expect “Extroversion”, one of the Big Five traits of human personality, to be implicated in the behavior of wearing a protective mask. Extroverted people are less likely to feel that they will be judged or evaluated by other people [25]. Consequently, we hypothesize that they may be more likely to wear protective mask despite of the potential social stigma. Our results reveal that wearing a facial mask is more common among individuals who are extroverted.

Another major contribution of this project is that we empirically identify the association between mask-wearing behavior and the social acceptability of this behavior. In their Spain-based study of cold and influenza, Ferng et al. [4] used focus group interviews to document that social acceptability seemed to be a barrier to mask-wearing. In this regard, many academics around the world have urged governments and citizens to make mask-wearing a universal social practice [26]. Ahmed, Harker and Edirisinghe [12] also argue that “in Europe and North America, there is stigmatisation for healthy people wearing facemasks which can cause racial aggravations, revised public education is therefore required” (10). The expectation is that universal use of masks will help overcome cultural barriers and lower the perceptions that the mask-wearers must be ill people. We contribute to this debate by empirically showing whether the social acceptability, as a descriptive social norm, influence the use of protective masks in our representative sample and, second, how the social context interacts with the effect of demographic characteristics, risk perceptions, and personality traits. In this regard, we show that an individual’s likelihood of mask-wearing is positively correlated with the proportion of uptake in the surrounding area. In addition, two key demographic factors, age and education, are importantly moderated by the proportion of uptake in the surrounding area.

## Methods

### Data collection

In this study, we conducted one of the first nationally representative surveys about this unprecedented public health emergency through a UK-based survey company, Respondi, which interviewed more than 4,000 Spanish individuals, who had opted-in for online surveys, with an approximate duration of 25 minutes each. They survey was responded in an online environment, and we obtained written consent from the participants of our survey. Respondents were remunerated for taking the time to complete the survey. The target group was individuals of Spanish nationality aged 18 or older, filling quotas in terms of gender, age categories, town size, and region. In total, we received 4,000 responses out of 5,500 survey invitations, which implies a 93% response rate. The high response rate is likely to be due to the online environment of the survey and the Spanish context at the time of the survey, with a stay-at-home order in place. Please see S1C Appendix for a full description of the demographic characteristics of respondents.

### Research context

**Fig 2:**
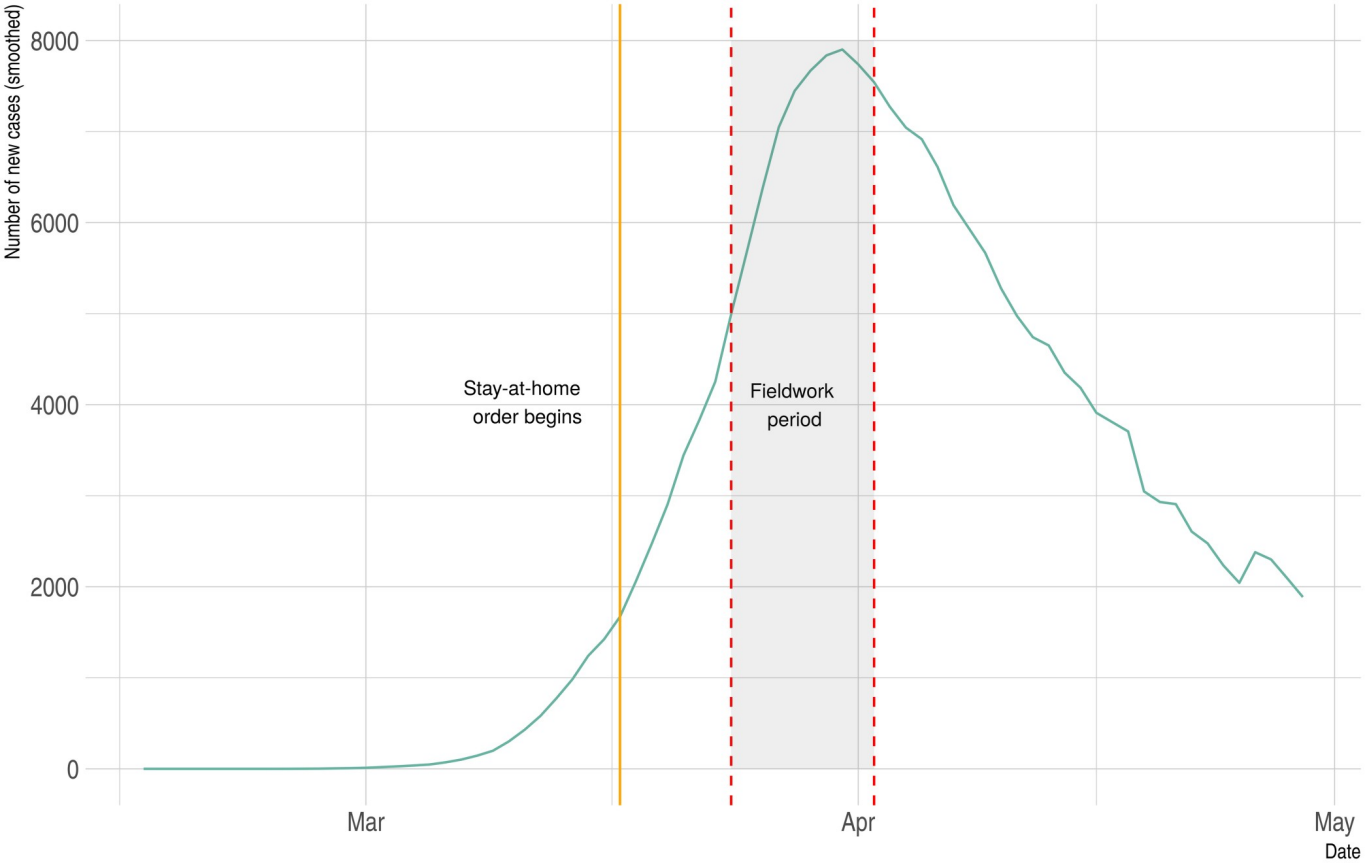
Distribution of new COVID-19 confirmed cases in Spain.

Fig 2 shows the evolution of the daily confirmed new cases of COVID-19 between February 15, 2020, and April 30, 2020. The dashed vertical lines indicate the fieldwork period, between March 24th and April 2nd, 2020. As we can observe in Fig 2, the number of daily cases was rapidly increasing during the survey fieldwork and it had not yet reached the peak of the first wave of infection. More specifically, 2,696 individuals had passed away and 39,673 individuals had been infected from the COVID-19 at the beginning of the fieldwork period. During this period, we should also note that the Spanish government was implementing the state of alarm and the near total lockdown of non-essential activities to prevent the spread of the COVID-19. These nation-wide policies went into effect on March 16th, 2020. Hence, respondents had been between a week and two weeks under lockdown by the time they were responding to the survey. At that time, however, the government had not yet implemented any policy with regards to mask-wearing behavior.

### Measures

The survey was designed to learn respondents’ mask-wearing behavior, and risk perceptions. Please refer to S1A Appendix for more details about the survey questions.

#### Dependent variable: Mask-wearing behavior

At the beginning of the survey, we asked the following question about respondents’ mask-wearing behavior: “After the outbreak of Novel Coronavirus (COVID-2019), have you worn a facemask?” with four items “Never”, “Rarely”, “Occasionally”, and “Very frequently”.

#### Independent variables

*Demographics*. For the set of demographics, we included gender, age group, and educational attainment.

*Risk perceptions*. We asked four questions: a) “How likely is that you have had or you currently have Coronavirus?” (from “Extremely unlikely” to “Extremely likely”); b) “How worried are you that you or someone in your family will be infected to the Coronavirus?” (from “Not worried at all” to “Very worried”); c) “What is your perceived likelihood of being infected with the Coronavirus in the future?” (from “Not possible at all” to “Very likely”) and, d) “No one wants to be infected with the Coronavirus, but if you are unfortunately infected, are you confident that you will be able to access to adequate medical care?” (from “Not confident at all” to “Very confident”).

*Personality traits*. Near the end of the survey, we added the Big-Five Inventory (BFI) validated in the Spanish language [27]. The BFI is a 10-item measure of the Big Five (or Five-Factor Model) personality domains, namely Extroversion, Openness, Conscientiousness, Agreeableness, and Emotional Stability (or Neuroticism).

*Social acceptability*. The survey allows us to geo-locate respondents in their province and region of residence. We compute the average in the use of face mask among all respondents who live in the same province (or region) of the respondent to generate a measure of social acceptability of the mask-wearing behavior. We exclude the same respondent from these computations to avoid measure endogeneity. In essence, this variable captures a descriptive norm of mask usage in the social context of the respondent.

#### Control variables

A major factor related to mask-wearing behavior has to do with respondents’ exposure to risky behaviors such as working and spending time outside home. We add three variables that capture how frequently people leave their homes and spend time in public. First, the models include dummies for 13 occupational categories: 1) business manager, 2) engineers, 3) medical workers (doctors and nurses), 4) teachers, 5) other qualified professionals, 6) middle skilled workers, 7) middle managers and supervisors, 8) technicians, 9) agriculture and industrial workers, 10) unskilled workers, 11) retired, 12) students, 13) unclassified and others. This variables should remove heterogeneity of exposure related to occupations.

Additionally, we control more directly for how much time respondents spend at home. The survey asks “After the outbreak of Novel Coronavirus (COVID-2019), have you stayed home more than usual?,” and the respondents could select “Definitely yes”, “Mostly yes”, “Mostly not”, and “Definitely not”. Note that the category “Not sure” was also an option and has been recoded as a missing value for analysis. Further, we also control for how the COVID had an impact on respondents’ job. The survey asks “Has your job been affected by the Coronavirus?,” and the respondents had the following options: “Yes, I have been affected by an ERTE”, “Yes, I have been dismissed”, “Yes, I have had to work from home”, and “No, I have not been affected”.

### Statistical analysis

Ordinal logistic regression analysis is used for estimating the associations between mask-wearing and our interested factors. We choose this estimator because our outcome variable–mask-wearing behavior– is ordinal and limited to four ordered categories: never, rarely, occasionally, very frequently. All statistical tables report ordinal logistic regression before and after controlling for control variables. Please refer to S1B Appendix for more details. All conclusions are robust to using linear models as shown in the S1D Appendix.

In the models where we explore the association between demographic characteristics and mask use by the prevalence of mask-wearing behavior in the province of residence, we follow two analytical paths. On the one hand, we split the sample in those who live in a context where the mask-wearing behavior is relatively prevalent (above the 60th percentile), and those who live in a context where the mask-wearing behavior is relatively less prevalent (below the 40th percentile). then, we run two separated ordinal logistic regressions, one for respondents who live in high-prevalent areas and another for those who live in low-prevalent areas. We report this split-model in the main manuscript where we can observe obvious differences in coefficients and significance levels depending on respondents’ context.

However, these models cannot ascertain whether differences in coefficients are statistically significant because models are not nested. Consequently, we complement the split-model with an interaction model where demographic predictors are all interacted with a dummy that indicates whether the respondent belongs to an area with a high prevalence of mask users. The p-values associated with the differences in coefficients by prevalence of mask use are reported together with the split-model to allow for an analytical evaluation of the moderating impact of context on the basic demographic relationships. For the sake of parsimony, the full interaction model is reported in the S1E Appendix.

### Ethics

This study was approved by the Human Research Committee of the New York University—Abu Dhabi in the United Arab Emirates (HRPP-2020-36). This project obtained a sample of respondents from Spain, a country different from the authors’ institution. We obtained the required permits and approvals to comply with the relevant national regulations and laws applying to research conducted by scholars in foreign institutions. Specifically, there is no formal IRB requirement for conducting social survey in Spain; however, we applied and obtained an IRB approval at New York University Abu Dhabi. As such, this research suffices global ethical standard.

## Results

**Fig 3:**
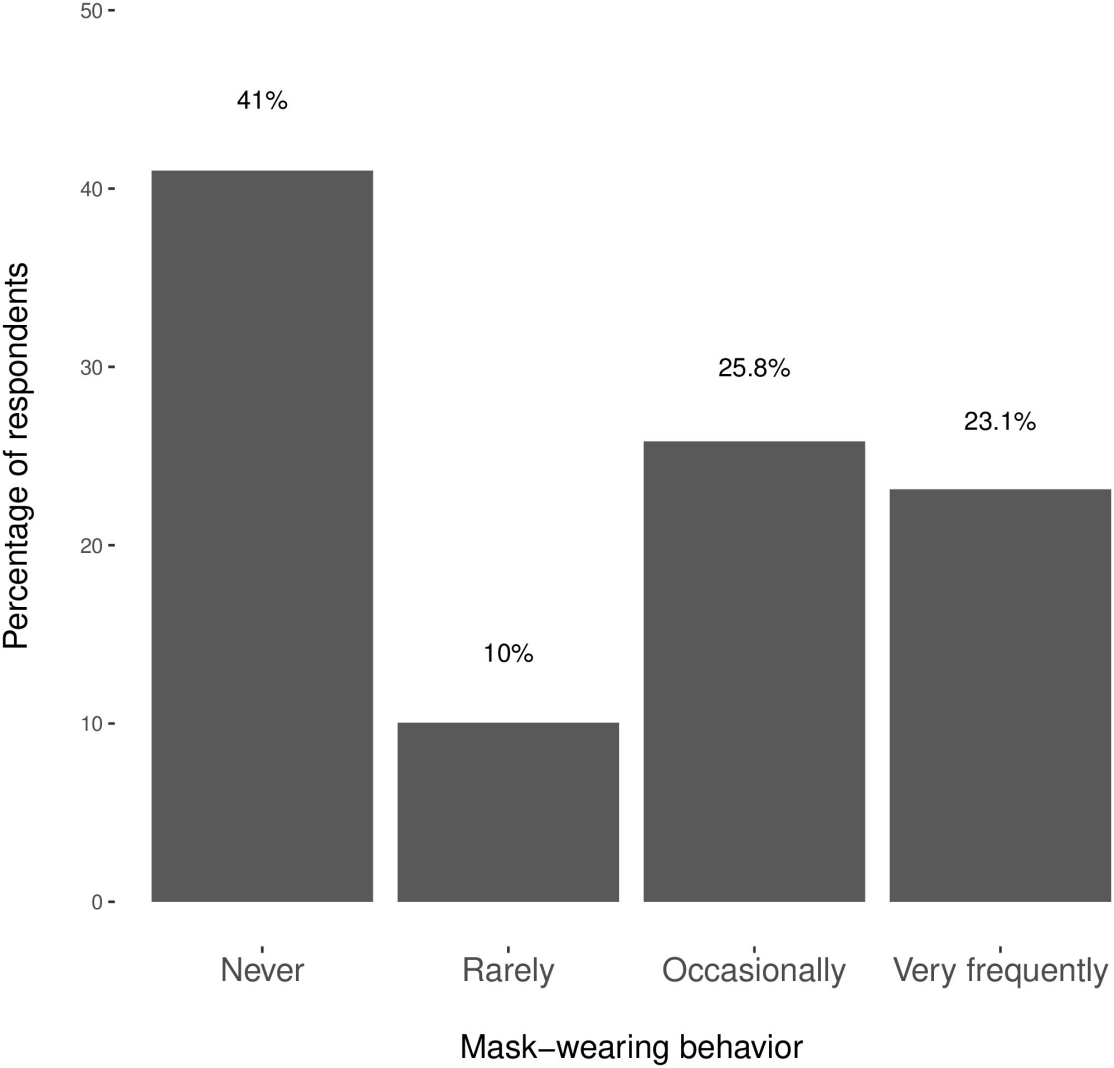
The prevalence of mask-wearing behavior in Spain during the COVID-19 outbreak.

We begin by describing the mask-wearing behavior of the Spanish population. Fig 3 shows that there are significant individual differences in the tendency to wear protective masks. While 48.9% of the respondents wear protective masks either occasionally or very frequently, 41% of the respondents admit to never wearing a facemask and 10% do so only rarely. Even though the COVID-19 death-rate in Spain was quickly climbing during our fieldwork period, surpassing that in nearly all other country, mask-wearing behavior remained far from universal. We now turn into investigating what explains these differences across individuals.

### Demographics

Table 1 shows the association between demographic characteristics and mask use. Ordinal logistic regression analysis revealed that wearing a face mask is significantly associated with age, education and occupation. The age cohort that is least likely to wear masks is the youngest (18-25). The subsequent age cohort (26-35) is significantly more likely to wear a mask. Further, every older age group is significantly more likely to wear a mask than the youngest cohort (18-25). Educational attainment is significantly associated with mask-wearing behavior. More specifically, college and graduate-educated people are significantly less likely wear a protective mask than the rest of the respondents. Finally, respondents who were inactive in the job market or could continue working remotely during the COVID-19 crisis were less likely to wear protective masks. S1D Appendix reports the results from the equivalent linear models.

**Table 1:** Ordinal logistic regressions investigating the association between demographic characteristics and mask use.

	DV: Wearing a face mask	
	OR (95% CI)	P Value
**Gender**		
Male		
Female	0.98 (0.87-1.11)	.78
**Age**		
Age: 18–25		
Age: 26–35	1.86 (1.40–2.48)	< .01
Age: 36–45	1.87 (1.48–2.37)	< .01
Age: 46–55	1.77 (1.39–2.24)	< .01
Age: 56–65	1.98 (1.55–2.53)	< .01
Age: 65+	2.26 (1.05–4.83)	.04
**Education**		
Secondary or lower		
High School	0.88 (0.73–1.07)	.19
Some college	0.91 (0.72–1.15)	.43
College	0.77 (0.63–0.94)	.01
Graduate school	0.71 (0.56–0.90)	< .01
**Time at home (last week)**	0.99 (0.85–1.14)	0.87
**COVID job impact**		
Inactive		
Dismissal	1.81 (1.18–2.76)	< .01
Temporary dismissal	1.96 (1.26–3.08)	< .01
Telework	1.13 (0.75–1.72)	.56
Not affected	1.85 (1.21–2.83)	< .01
**Occupation Fixed Effects**	Yes	Yes
N	3,906	
AIC	9,846	

Some of these associations are comparable to those in the literature. Evidence on the mask-wearing behavior during an early phase of the H1N1 influenza epidemic in Honk Kong revealed that female respondents were more willing to wear face masks [17]. In three samples from the United States, Jordan et al., Capraro and Barceló [15], and Haischer et al. [16] also find that women report lower intentions to wear a face covering and are, thus, less likely to wear it. In contrast to these findings, we find no gender effect in Spain. Further research is needed to understand the gender patterns of wearing protective masks, and its cross-national variation.

### Risk perceptions concerning the COVID-19

Table 2 shows an ordinal logistic regression with predictors of risk perceptions unadjusted for demographics (column 1) and adjusted for demographics (column 2). These models reveal that those respondents who believe that they are neither likely nor unlikely to be currently infected, likely to be currently infected, or very likely to be currently infected, and those who are somewhat concerned about being infected themselves or a close family member or very concerned about being infected themselves or a close family member are significantly more likely to comply with the mask-wearing behavior. By contrast, respondents who perceive that they are very likely to be infected in the future are significantly less likely to wear a protective mask. Finally, trust in the health system does not seem to exert a systematic effect on mask-wearing behavior.

**Table 2:** The association between risk perceptions and mask use.

	DV: Wearing a face mask			
	OR (95% CI)	P Value	OR (95% CI)	P Value
**Likely to be infected now**				
Very unlikely				
Unlikely	1.00 (0.85-.117)	.97	0.98 (0.83-1.16)	.81
Neither likely nor unlikely	1.33 (1.15-1.54)	< .01	1.25 (1.07-1.46)	< .01
Likely	1.54 (1.17-2.02)	< .01	1.52 (1.14-2.02)	< .01
Very likely	3.40 (2.11-5.54)	< .01	2.91 (1.76-4.84)	< .01
**Concerned about becoming infected**				
Not concerned at all				
Not concerned	1.32 (0.81-2.17)	.28	1.26 (0.75-2.15)	.38
Somewhat concerned	1.71 (1.11-2.69)	.02	1.62 (1.02-2.62)	.04
Very concerned	2.58 (1.68-4.05)	< .01	2.49 (1.57-4.01)	< .01
**Likely to be infected in the future**				
Very unlikely				
Unlikely	0.84 (0.53-1.31)	.43	0.68 (0.42-1.10)	.11
Neither likely nor unlikely	0.92 (0.58-1.45)	.70	0.69 (0.42-1.13)	.14
Likely	0.89 (0.57-1.40)	.61	0.75 (0.46-1.22)	.24
Very likely	0.72 (0.45-1.17)	.18	0.59 (0.35-0.99)	.04
**Trust in health system**				
Do not trust				
Tend not to trust	1.47 (1.03-2.10)	.04	1.47 (1.02-2.14)	.04
Tend to trust	1.31 (0.93-1.85)	.13	1.26 (0.89-1.81)	.20
Trust	1.34 (0.95-1.90)	.10	1.28 (0.90-1.84)	.18
**Controls**				
Demographics?	No		Yes	
Occupation Fixed Effects?	No		Yes	
Time at home?	No		Yes	
COVID job impact?	No		Yes	
N	3,958		3,769	
AIC	10,075		9,437	

Overall, we find that the public exhibit a greater likelihood of wearing a mask when their perceived risk of being affected by the outbreak is high or they have a significant level of disease-related distress. This is consistent with previous research across a number of countries, including Norway [18], United Kingdom [19], and Hong Kong [17], that found that risk perceptions influence the intentions to wear a face covering to prevent the transmission of other respiratory illnesses such as the H1N1 influenza and the swine flu. Further, it is consistent with Jordan et al.’s [14] findings during the COVID-19 pandemic in their convenient sample of American citizens.

### Personality traits

Table 3 reports ordinal logistic regressions that associate personality traits with mask use unadjusted for demographics (column 1) and adjusted for demographics (column 2). Both columns show that extroverted respondents are more likely to wear a mask than introverted respondents. This is the only personality trait that shows a systematic association with mask-wearing behavior.

**Table 3:** The association between personality traits and mask use.

	DV: Wearing a face mask			
	OR (95% CI)	P Value	OR (95% CI)	P Value
**Personality traits**				
Extroversion	1.08 (1.05-1.10)	< .01	1.06 (1.04-1.09)	< .01
Openness to new experiences	0.99 (0.96-1.02)	.46	1.00 (0.97-1.03)	.82
Agreeableness	1.01 (0.97-1.05)	.60	1.02 (0.98-1.06)	.41
Conscientiousness	1.00 (0.98-1.03)	.78	1.00 (0.97-1.03)	.94
Neuroticism	0.99 (0.96-1.01)	.33	0.99 (0.97-1.02)	.66
**Controls**				
Demographics?	No		Yes	
Occupation Fixed Effects?	No		Yes	
Time at home?	No		Yes	
COVID job impact?	No		Yes	
N	3,948		3,902	
AIC	10,128		9,826	

### Social acceptability

Table 4 reports ordinal logistic regressions that link the social acceptability of the mask-wearing behavior in a respondent’s social context and individual mask-wearing behavior after adjusting or not for demographic characteristics. The models reveals that respondents are more likely to wear a mask if they live in a region or a province where mask-wearing behavior is common. By contrast, the actual number of infected cases in a province or a region is not systematically associated with using a mask. The significant effect of social acceptability upholds after controlling for the number of infected cases in the region or province and adding fixed effect by survey day.

**Table 4:** The association between social acceptability and mask use.

Panel A: Regional Level	DV: Wearing a face mask			
	OR (95% CI)	P Value	OR (95% CI)	P Value
**Social Acceptability**				
Regional average of mask users	2.90 (2.01-4.19)	< .01	3.23 (2.19-4.79)	< .01
*log* Positive cases in the region (per 100,000 inhabitants)	0.99 (0.91-1.08)	.88	0.76 (0.28-2.10)	.59
Demographics?	No		Yes	
Occupation Fixed Effects?	No		Yes	
Time at home?	No		Yes	
COVID job impact?	No		Yes	
Survey date FE	Yes		Yes	
N	4,132		3,901	
N Regions	18		18	
AIC	10,602		9,815	
Panel B: Province Level	DV: Wearing a face mask			
	OR (95% CI)	P Value	OR (95% CI)	P Value
**Social Acceptability**				
Provincial average of mask users	2.34 (1.78-3.08)	< .01	2.41 (1.82-3.20)	< .01
*log* Positive cases in the province (per 100,000 inhabitants)	1.01 (0.93-1.09)	.84	1.01 (0.94-1.10)	.60
Demographics?	No		Yes	
Occupation Fixed Effects?	No		Yes	
Time at home?	No		Yes	
COVID job impact?	No		Yes	
Survey date FE	Yes		Yes	
N	3,934		3,893	
N Provinces	52		52	
AIC	10,091		9,782	

Further, Table 5 reports the split-sample models where we identify personal characteristics of mask-wearing behavior that interact with the social norms to wear masks. The last column in Table 5 reports the difference in coefficients that results from the interaction model, which is reported in S1E Appendix.

**Table 5:** The association between demographic characteristics and mask use by the prevalence of mask-wearing behavior in the province.

	DV: Wearing a face mask				
	Low prevalence of masks		High prevalence of masks		Diff.
	OR (95% CI)	P Value	OR (95% CI)	P Value	P Value
**Gender**					
Male					
Female	0.88 (0.71-1.08)	0.21	0.97 (0.80-1.18)	0.76	.49
**Age**					
Age: 18-25					
Age: 26-35	2.15 (1.34-3.47)	< .01	2.13 (1.32-3.42)	< .01	.97
Age: 36-45	1.55 (1.03-2.34)	.03	2.90 (1.94-4.35)	< .01	.03
Age: 46-55	1.45 (0.97-2.20)	.07	2.62 (1.76-3.93)	< .01	.04
Age: 56-65	1.34 (0.87-2.07)	.19	2.86 (1.92-4.28)	< .01	< .01
Age: 65+	0.87 (0.21-3.27)	.84	6.33 (2.22-19.12)	< .01	.02
**Education**					
Secondary or lower					
High School	0.67 (0.49-0.91)	.01	1.14 (0.85-1.53)	.37	.01
Some college	0.68 (0.46-1.01)	.06	1.16 (0.82-1.64)	.39	.05
College	0.61 (0.44-0.83)	< .01	0.90 (0.66-1.22)	.50	.09
Graduate school	0.72 (0.48-1.06)	.10	0.73 (0.51-1.07)	.10	.96
**Personality traits**					
Extroversion	1.06 (1.02-1.10)	< .01	1.08 (1.04-1.12)	< .01	0.40
Openness	1.02 (0.98-1.07)	.32	0.97 (0.93-1.02)	.26	0.13
Agreeableness	1.02 (0.96-1.09)	.53	1.00 (0.94-1.07)	.95	0.70
Conscientiousness	1.01 (0.96-1.06)	.70	1.00 (0.96-1.05)	.84	0.89
Neuroticism	1.00 (0.96-1.04)	.97	0.99 (0.95-1.03)	0.52	0.67
**Province infection rate**					
*log* Positive cases (per 100,000 inhabitants)	1.87 (1.64-2.13)	< .01	0.98 (0.87-1.11)	.76	< .01
Survey date FE	Yes		Yes		
N	1,552		1,762		
N Provinces	52		52		

*Note*: Column Diff. shows the P-Value of the difference in coefficients between the subgroups, high and low prevalence of the mask-wearing behavior in respondents’ province of residence, at the 95% confidence level, based on a fully-specified interaction model. All models control for occupation fixed effects, time spent at home in the last week, and the impact of the COVID on respondents’ employment status. See S1E Appendix for the full output of the interaction model.

The results reveal that older people tend to follow the socially-accepted behavior in their area of residence. In a social context where wearing a protective mask is relatively less common, older age groups are no more likely to wear a mask than younger age groups. By contrast, respondents in the youngest cohorts are the least likely to wear a protective mask in a context where mask-wearing behavior is common. In this context, the subsequent age cohort (26-35) is significantly more likely to wear a mask than the youngest cohort. The effect of age becomes especially strong for the 36-45 age group, and all subsequent age groups; namely, those aged 46-55, 56-65, and 65+. While there are some differences across cohorts in all social contexts, the difference of mask-wearing across cohorts is much stronger in areas where wearing a face mask is prevalent.

The interaction model (reported in S1E Appendix) shows that the impact of social context on the coefficients of the age groups *36-45*, *46-55*, *56-65*, and *65+* are statistically significant at the 95% confidence level. In the literature, Taylor et al. [28] analyzed the 2007 New South Wales population health survey in Australia and found that younger people (16-24) were the least willing to comply with face mask wearing among age groups. Digging deeper, we can see the substantial age effect is originating from the provinces with higher prevalence of maskwear-ing, this result is likely because the elderly are more affected by social norms. That is, senior cit-izens are more willing to follow what other people are doing.

Educational attainment is negatively associated with wearing a face-mask in areas where mask-wearing behavior is less common. In low-prevalent environments, the least-educated are more likely to wear a face mask compared to those who hold a high school diploma, have attended some college, have completed a college degree, or have attended graduate school. Conversely, strong social norms of mask-wearing behavior make respondents with a high school diploma, some college, and a college degree no less likely to wear a mask than those who have a secondary degree or lower. By contrast, respondents who have attended graduate school remain resistant to social norms and, thus, continue being marginally less likely to the use of protective masks even in highly prevalent environments.

Previously, Lau et al. [17] found those who attained a higher education level were more likely to wear face masks during the H1N1 influenza pandemic in Hong Kong. This contrast may arise because, unlike in the Hong Kong context, there are significant contradicting opinions among medical experts and government officials about the necessity to wear a facial mask in Spain. Higher education encourages critical thinking, and hence highly educated but skeptical members of the public are less likely to naively follow government recommendation of mask-wearing when there are contradicting opinions provided by medical experts. While most resistance of mask-wearing from highly educated citizens would be reconciled with strong social norms of mask-wearing behavior, those who have attended graduate school tend to continue denying mask-wearing even if they live in an area of high acceptability of mask use. This result indicates that when the government is encouraging mask-wearing, solid arguments—in conjunction and alignment with advice form medical experts—are necessary to convince their highly educated citizens.

The personality trait of Extroversion is significantly associated with mask-wearing behavior regardless of the prevalence of that behavior. Extroverted people are more likely to wear a protective mask in both areas where relatively fewer people wearing a mask and areas where relatively more people frequently wear a mask. The interaction model (reported in S1E Appendix) shows that the impact of social context on these coefficients is not statistically significant at standard confidence levels.

Finally, the lack of an overall association between the infection rate in the province of residence and mask-wearing behavior (see Table 4) masks significant heterogeneous effects. Where wearing a mask is relatively rare, respondents who live in virus-affected areas are more likely to wear a protective mask. In areas where wearing a mask is a common behavior, the number of cases in the province does not significantly influence respondents’ mask-wearing behavior. The interaction model (reported in S1E Appendix) shows that the impact of social context on these coefficients is statistically significant at the 99% confidence level.

## Conclusions

As we are writing, more countries without previous culture of mask-wearing are recommending their citizens to voluntarily wear facial masks. It is, thus, imperative for the government to identify the barriers to mask-wearing in order to devise effective programs for improving public compliance. Our study, analysing the data from one of the first nationally representative surveys (n = 4,000) in Spain, confirms some previously identified determinants of individual variation of wearing a facial mask, and uncovers many that have never been documented before.

We established the associations of mask-wearing with factors, such as age, education level, personality traits, risk-perception, and social acceptability of mask-wearing. Two main contributions of this paper are to: 1) empirically investigate the role of the Big Five in wearing facial masks, and demonstrates that wearing a facial mask is more common among individuals who are extroverted; and 2) provide empirical evidence for the positive association between mask-wearing behavior and the social acceptability of this behavior. We further explored the interaction effects between these factors. The results are robust to alternative specifications, including controlling for respondents’ occupation and time spent in public.

Our findings offer both policy and theoretical contributions. From a policy perspective, we offer practical suggestions for governments that would like to persuade their citizens to wear face-masks in public settings. First, we provide a profile of citizens who are more resistant to the uptake of face-mask when their use voluntary: young, educated, and unconcerned with being infection. Thus, governments should consider to calibrate persuasive appeals to these social groups. Second, psychological research has shown that psychological targeting can be an effective tool of persuasion when the personality traits of the target audience is known [29]. Our findings show that introverted are the least likely to wear a face-mask when wearing a mask lacks cultural roots and is not mandatory. Therefore, government should also consider to generate persuasive appeals that are psychologically targeted to introverts. Third, our results indicate a positive correlation between social acceptability and mask uptake, and demonstrate that those people, and especially elderly people, who live in high-prevalent areas are more likely to use it compared to those who live in low-prevalent areas. This finding suggests that government should consider to creatively use marketing materials to shape people’s beliefs of social norms around where they live to encourage the voluntary uptake of face-masks.

From a theoretical perspective, we establish the systematic association between mask-wearing behavior and sociological and psychological factors by making several contributions to the current debates. First, these findings contribute to a growing literature that tries to identify the determinants of mask-wearing behavior amidst the COVID-19 global crisis [14–16], and other pandemics [4, 17–19]. Beyond studies of mask-wearing behavior, our findings also speak to a broader literature on the social, economic, and political determinants of compliance with health-preventive behavior during the COVID-19 pandemic around the world [30–33].

Further, we also make a theoretical contribution to the sociological literature on contextual or neighborhood effects. A long body of scholarship argues that local normative contexts shape people’s behavior beyond what one would expect from the characteristics of the individuals alone, which suggests an independent and unique effect of the environment. Prior work identified this neighborhood effect in a wide array of study areas, including policy preferences [34], community engagement [35], social identity [36–38], educational outcomes, and health status [39]. We contribute to this literature by demonstrating how social norms in the immediate environment shape the voluntary adoption of protective masks during a pandemic.

We acknowledge that there are at least three limitations that should be considered when assessing the implications of this paper. First, we recognize that different respiratory diseases pertain different levels of necessity of mask-wearing. Thus, while due to the scarcity of related literature, we compare our results with previous studies on all respiratory diseases, including flu [4, 11, 17], we are aware that these results are not perfectly comparable, because people might act differently when facing different diseases. To learn a more precise individual behavioral pattern, more research during outbreaks of diseases of different severity are essential. Second is external validity, as already discussed, some of our findings from the nationally representative survey of Spain are not consistent with findings from other countries, this means some of our results may not be directly applicable to other countries. For policy makers to devise effective programs for improving public compliance with mask-wearing, country- or region-specific surveys are necessary. The possible determinants we highlighted in this study should be taken into account. Third, we analyze self-reported behavior survey in this paper, and thus social desirability bias can potentially be an issue. However, considering that, when the survey was fielded, mask-wearing was not officially recommended by most Western countries, and Spain has no culture of wearing masks, we believe the bias to be minimal.

## Supporting information

S1 Appendix (PDF)
