# Supplementary material for: Voluntary adoption of social welfare-enhancing behavior: Mask-wearing in Spain during the COVID-19 outbreak
Source: PLoS One. 2020 Dec 1;15(12):e0242764. doi: 10.1371/journal.pone.0242764 (PMC7707551; doi:10.1371/journal.pone.0242764)
Supplement: S1 Appendix — (PDF) [file pone.0242764.s001.pdf]

S1 Appendix

A Data collection

A.1 Survey questions

For the set of demographics, we included gender, age group, and educational attainment.

We asked four questions: a) “How likely is that you have had or you currently have Coronavirus?” (from “Extremely unlikely” to “Extremely likely”); b) “How worried are you that you or someone in your family will be infected to the Coronavirus?” (from “Not worried at all” to “Very worried”); c) “What is your perceived likelihood of being infected with the Coronavirus in the future?” (from “Not possible at all” to “Very likely”) and, d) “No one wants to be infected with the Coronavirus, but if you are unfortunately infected, are you confident that you will be able to access to adequate medical care?” (from “Not confident at all” to “Very confident”).

Near the end of the survey, we added the Big-Five Inventory (BFI) validated in the Spanish language [27]. The BFI is a 10-item measure of the Big Five (or Five-Factor Model) personality domains, namely Extroversion, Openness, Conscientiousness, Agreeableness, and Emotional Stability (or Neuroticism).

The survey allows us to geo-locate respondents in their province and region of residence. We compute the average in the use of face mask among all respondents who live in the same province (or region) of the respondent to generate a measure of social acceptability of the mask-wearing behavior. Note that we exclude the same respondent from these computations to avoid measure endogeneity. In essence, this variable captures a descriptive norm of mask usage in the social context of the respondent.

Occupational categories are collapsed in 13 categories: 1) business manager (combines “Big businesses” and “Small businesses” were asked independently in the survey but they are combined in the analyses because each gathered few responses), 2) engineers, 3) medical workers (combines “Doctors” and “Nurses” were asked independently in the survey but they are combined in the analyses because each gathered few responses), 4) teachers, 5) other qualified professionals, 6) middle skilled workers, 7) middle managers and supervisors (“Middle Managers” and “Supervisors” were asked independently in the survey but they are combined in the analyses because each gathered few responses), 8) technicians, 9) agriculture and industrial workers (combines “Agriculture workers” and “Industrial workers” were asked independently in the survey but they are combined in the analyses because each gathered few responses), 10) unskilled workers, 11) retired (indirectly built through a combination of age above 65 and a missing value in occupation), 12) students (indirectly built through a combination of age below 25 and a missing value in occupation), 13) unclassified and others.

Additionally, we control more directly for how much time respondents spend at home. The survey asks “After the outbreak of Novel Coronavirus (COVID-2019), have you stayed home more than usual?,” and the respondents could select “Definitely yes”, “Mostly yes”, “Mostly not”, and “Definitely not”. Note that the category “Not sure” was also an option and has been recoded as a missing value for analysis. Further, we also control for how the COVID had an impact on respondents’ job. The survey asks “Has your job been affected by the Coronavirus?”, and the respondents had the following options: “Yes, I have been affected by an ERTE”, “Yes, I have been dismissed”, “Yes, I have had to work from home”, and “No, I have not been affected”.

B Methods

Ordinal logistic regression analysis was used to explore statistical associations between wearing a face mask and the following factors: 1) Demographic characteristics, 2) Risk perceptions concerning the COVID-19, 3) Personality traits, and 4) Social acceptance of the mask-wearing behavior. Results were displayed in the log odds scale with 95% Confidence Intervals (95% CI). All analysis were performed using **R** (version 3.6.3). For the social acceptance models we estimated clustered standard errors to account for the within-region and the within-province correlation across observation in the independent variables.

We computed the average in the use of face mask among all respondents who live in the same region and province of the respondent to generate a measure of social acceptability of the mask-wearing behavior. To avoid endogeneity in the measure, we exclude the same respondent from these computations.

C Descriptive statistics

Table C.1. Demographic characteristics of respondents

Characteristics	Proportions	
**Gender**	Proportion	N
Male	49.6%	1,939
Female	50.4%	1,967
All	100%	3,906
**Age**		
Age: 18-25	10.2%	398
Age: 26-35	14.4%	561
Age: 36-45	18.9%	737
Age: 46-55	18.8%	736
Age: 56-65	15.4%	601
Age: 65+	22.3%	873
All	100%	3,906
**Occupation**		
Secondary or lower	14.3%	557
High School	27.0%	1,053
Some college	12.0%	470
College	31.8%	1,244
Graduate school	14.9%	582
All	100%	3,906
**Occupation**		
Other	97.8%	3,820
Physician	0.8%	30
Nurse	1.4%	56
All	100%	3,906

D Linear models

D.1 Demographics

Table D.1. Linear Regressions Investigating the Association Between Demographic Characteristics and Mask Use

	DV: Wearing a face mask	
	β (95% CI)	P Value
**Gender**		
Male		
Female	0.00 (-0.08, 0.08)	.92
**Age**		
Age: 18–25		
Age: 26–35	0.41 (0.23, 0.59)	$< .01$
Age: 36–45	0.42 (0.27, 0.57)	$< .01$
Age: 46–55	0.39 (0.24, 0.54)	$< .01$
Age: 56–65	0.47 (0.31, 0.63)	$< .01$
Age: 65+	0.52 (0.06, 0.99)	.03
**Education**		
Secondary or lower		
High School	-0.08 (-0.20, 0.05)	.22
Some college	-0.06 (-0.21, 0.09)	.41
College	-0.16 (-0.28, -0.03)	.01
Graduate school	-0.21 (-0.37, -0.06)	.01
**Time at home (last week)**	0.00 (-0.09, 0.09)	1.00
**COVID job impact**		
Inactive		
Dismissal	0.40 (0.13, 0.68)	$< .01$
Temporary dismissal (ERTE)	0.44 (0.15, 0.73)	$< .01$
Telework	0.09 (-0.18, 0.36)	.50
Not affected	0.41 (0.14, 0.69)	$< .01$
**Occupation Fixed Effects**	Yes	Yes
N	3,906	

D.2 Risk perceptions

Table D.2. Linear Regressions of the Association Between Risk Perceptions and Mask Use

	DV: Wearing a face mask			
	β (95% CI)	P Value	β (95% CI)	P Value
**Likely to be infected now**				
Very unlikely				
Unlikely	0.01 (-0.10, 0.11)	0.92	0.00 (-0.11, 0.10)	.97
Neither likely nor unlikely	0.19 (0.09, 0.28)	< .01	0.14 (0.04, 0.23)	< .01
Likely	0.28 (0.10-0.46)	< .01	0.25 (0.07, 0.43)	.01
Very likely	0.75 (0.44, 1.05)	< .01	0.60 (0.30, 0.90)	< .01
**Concerned about becoming infected**				
Not concerned at all				
Not concerned	0.16 (-0.14,0.46)	.31	0.12 (-0.19, 0.43)	.43
Somewhat concerned	0.31 (0.04, 0.58)	.02	0.27 (-0.01, 0.54)	.06
Very concerned	0.59 (0.32, 0.85)	< .01	0.54 (0.26, 0.81)	< .01
**Likely to be infected in the future**				
Very unlikely				
Unlikely	-0.12 (-0.40, 0.16)	.42	-0.25 (-0.54, 0.05)	.10
Neither likely nor unlikely	-0.06 (-0.34, 0.22)	.68	-0.23 (-0.53, 0.07)	.13
Likely	-0.08 (-0.36, 0.20)	.58	-0.18 (-0.48, 0.12)	.23
Very likely	-0.21 (-0.51, 0.09)	.17	-0.32 (-0.64, -0.01)	.04
**Trust in health system**				
Do not trust				
Tend not to trust	0.24 (0.01, 0.46)	.04	0.22 (-0.01, 0.44)	.06
Tend to trust	0.16 (-0.06, 0.37)	.15	0.12 (-0.10, 0.34)	.27
Trust	0.17 (-0.04, 0.39)	.12	0.13 (-0.09, 0.35)	.25
**Controls**				
Demographics?	No		Yes	
Occupation Fixed Effects?	No		Yes	
Time at home?	No		Yes	
COVID job impact?	No		Yes	
N	3,958		3,769	

D.3 Personality traits

Table D.3. Linear Regressions of the Association Between Personality Traits and Mask Use

	DV: Wearing a face mask			
	β (95% CI)	P Value	β (95% CI)	P Value
**Personality traits**				
Extroversion	0.05 (0.03, 0.06)	< .01	0.04 (0.02, 0.05)	< .01
Openness to new experiences	-0.01 (-0.03, 0.01)	.41	0.00 (-0.02, 0.02)	.73
Agreeableness	0.01 (-0.02, 0.03)	.62	0.01 (-0.02, 0.03)	.45
Conscientiousness	0.00 (-0.01, 0.02)	.68	0.00 (-0.02, 0.02)	.83
Neuroticism	-0.01 (-0.03, 0.01)	.34	0.00 (-0.02, 0.01)	.66
**Controls**				
Demographics?	No		Yes	
Occupation Fixed Effects?	No		Yes	
Time at home?	No		Yes	
COVID job impact?	No		Yes	
N	3,948		3,902	
AIC	10,128		9,826	

D.4 Social acceptability

Table D.4. Linear Regressions of the Association Between Social Acceptability and Mask Use

Panel A: Regional Level	DV: Wearing a face mask			
	β (95% CI)	P Value	β (95% CI)	P Value
**Social Acceptability**				
Regional average of mask users	2.90 (2.01-4.19)	< .01	3.23 (2.19-4.79)	< .01
log Positive cases in the region (per 100,000 inhabitants)	0.99 (0.91-1.08)	.88	0.76 (0.28-2.10)	.59
Demographics?	No		Yes	
Occupation Fixed Effects?	No		Yes	
Time at home?	No		Yes	
COVID job impact?	No		Yes	
Survey date FE	Yes		Yes	
N	4,132		3,901	
N Regions	18		18	
AIC	10,602		9,815	

Panel B: Province Level	DV: Wearing a face mask			
	OR (95% CI)	P Value	OR (95% CI)	P Value
**Social Acceptability**				
Provincial average of mask users	2.34 (1.78-3.08)	< .01	2.41 (1.82-3.20)	< .01
log Positive cases in the province (per 100,000 inhabitants)	1.01 (0.93-1.09)	.84	1.01 (0.94-1.10)	.60
Demographics?	No		Yes	
Occupation Fixed Effects?	No		Yes	
Time at home?	No		Yes	
COVID job impact?	No		Yes	
Survey date FE	Yes		Yes	
N	3,934		3,893	
N Provinces	52		52	
AIC	10,091		9,782	

E Interaction model

Table E.1. The Association Between Demographic Characteristics and Mask Use by the Prevalence of Mask-wearing Behavior in the Province. Interaction Model.

	DV: Wearing a face mask		
	Coefficient	95% CI	P-Value
Area with High Prevalence of Masks	2.40	(−0.43, 5.23)	.10
Female	−0.13	(−0.33, 0.08)	.22
Age: 26-35	0.75	(0.28, 1.23)	.<.01
Age: 36-45	0.43	(0.03, 0.84)	.04
Age: 46-55	0.37	(−0.04, 0.78)	.08
Age: 56-65	0.29	(−0.14, 0.72)	.19
Age: 65+	−0.14	(−1.48, 1.20)	.84
Education: High School	−0.40	(−0.71,−0.09)	0.01
Education: Some College	−0.38	(−0.77,0.01)	.06
Education: College	−0.49	(−0.81,−0.17)	<.01
Education: Graduate School	−0.33	(−0.72,0.06)	.10
Extroversion	0.06	(0.02, 0.10)	<.01
Openness	0.02	(−0.02,0.07)	.32
Agreeableness	0.02	(−0.04,0.08)	.53
Conscientiousness	0.01	(−0.04,0.05)	.71
Neuroticism	−0.001	(−0.04,0.04)	.97
Time at home	−0.08	(−0.31,0.16)	.52
Provincial number of infected cases (log)	0.61	(0.49,0.74)	<0.01
COVID Job Impact	Yes		
FE Occupation	Yes		
FE date	Yes		
High × Female	0.10	(−0.18,0.38)	.49
High × Age: 26-35	0.01	(−0.66,0.68)	.97
High × Age: 36-45	0.65	(0.07,1.22)	.03
High × Age: 46-55	0.61	(0.04,1.18)	.04
High × Age: 56-65	0.78	(0.19,1.37)	<.01
High × Age: 65+	2.01	(0.30,3.72)	.01
High × Education: High School	0.53	(0.11,0.96)	.01
High × Education: Some College	0.53	(0.01,1.06)	.05
High × Education: College	0.38	(−0.05,0.82)	.09
High × Education: Graduate School	0.02	(−0.53,0.56)	.96
High × Extroversion	0.06***	(0.02,0.10)	.39
High × Openness	−0.05	(−0.11,0.02)	.13
High × Agreeableness	−0.02	(−0.10,0.07)	.69
High × Conscientiousness	−0.004	(−0.07,0.06)	.89
High × Neuroticism	−0.01	(−0.07,0.05)	.67
High × Time at home	0.09	(−0.24,0.42)	.59
High × Provincial number of infected cases (log)	−0.63	(−0.81,−0.46)	<.01
High × COVID Job Impact	Yes		
High × FE Occupation	Yes		
High × FE date	Yes		

Note: This model only includes observations who live in areas with a prevalence of mask-wearing behavior of 40% or below (*Low*, low prevalence areas) and 60% or above (*High*, high prevalence areas) ($n = 3,318$).
